# Predicting antimicrobial peptides with improved accuracy by incorporating the compositional, physico-chemical and structural features into Chou’s general PseAAC

**DOI:** 10.1038/srep42362

**Published:** 2017-02-13

**Authors:** Prabina Kumar Meher, Tanmaya Kumar Sahu, Varsha Saini, Atmakuri Ramakrishna Rao

**Affiliations:** 1 Division of Statistical Genetics, ICAR-Indian Agricultural Statistics Research Institute, New Delhi-110012, India; 2 Centre for Agricultural Bioinformatics, ICAR-Indian Agricultural Statistics Research Institute, New Delhi-110012, India; 3 Department of Bioinformatics, Janta Vedic College, Baraut, Baghpat-250611, Uttar Pradesh, India

## Abstract

Antimicrobial peptides (AMPs) are important components of the innate immune system that have been found to be effective against disease causing pathogens. Identification of AMPs through wet-lab experiment is expensive. Therefore, development of efficient computational tool is essential to identify the best candidate AMP prior to the *in vitro* experimentation. In this study, we made an attempt to develop a support vector machine (SVM) based computational approach for prediction of AMPs with improved accuracy. Initially, compositional, physico-chemical and structural features of the peptides were generated that were subsequently used as input in SVM for prediction of AMPs. The proposed approach achieved higher accuracy than several existing approaches, while compared using benchmark dataset. Based on the proposed approach, an online prediction server *i*AMPpred has also been developed to help the scientific community in predicting AMPs, which is freely accessible at http://cabgrid.res.in:8080/amppred/. The proposed approach is believed to supplement the tools and techniques that have been developed in the past for prediction of AMPs.

Antimicrobial peptides (AMPs) are important innate immune molecules, which have been found to be effective against several pathogenic micro-organisms like bacteria, virus, fungi, parasites etc^1^. AMP constitutes the first line of host defense against microbes^2^, where it causes the cell death of microbes either by disrupting its cell membrane or its intracellular functions^3^,^4^. Due to growing resistance of microbial pathogens against chemical antibiotics, AMPs have received attention as an alternative in recent years^5^. Specifically, due to the broad spectrum of activity and low propensity for developing resistance, AMPs are gaining popularity in clinical applications^6^.

Development of sequence-based computational tools can be helpful in designing the effective antimicrobial agents by identifying the best candidate AMP prior to the synthesis and testing against pathogens in wet-lab^7^. In this direction, computational tools like AntiBP^1^, AMPER^8^, CAMP^3^, AntiBP2^9^, AVPpred^10^, ClassAMP^11^, iAMP-2L^7^ and EFC-FCBF^12^ have been developed for the prediction of AMPs. The binary (0, 1) and compositional features were used in AntiBP and AntiBP2 respectively to map the peptide sequences onto numeric feature vectors, where the numeric vectors were used as input in artificial neural network (ANN)^13^ and support vector machine (SVM)^14^ respectively for prediction of antibacterial peptides. In CAMP, random forest (RF)^15^, SVM and ANN supervised learning techniques were employed for prediction of AMPs, based on different physico-chemical (PHYC) features of peptides. In AVPpred, four different models viz., AVPmotif, AVPalign, AVMcompo and AVPphysico were developed for prediction of antiviral peptides only. The ClassAMP^11^ tool was developed for predicting the propensity of a peptide sequence as antibacterial, antiviral or antifungal peptide, by using SVM and RF machine learning techniques. In an another study, a two-level multi-class predictor was developed for identification of AMPs, based on Chou’s pseudo amino acid composition^16^ and fuzzy k-nearest neighbor^7^. Recently, Veltri *et al*.^12^ have developed a machine learning based computational approach for improved recognition of AMPs.

The above mentioned methods have their own advantages in generating knowledge for the prediction of AMPs. However, further improvement in prediction accuracy is required to minimize the number of false positives. In this study, we made an attempt to develop a computational approach for prediction of antibacterial, antiviral and antifungal peptides with higher accuracy. In this approach, combinations of compositional, PHYC and structural (STRL) features were used to map the peptide sequences onto numeric feature vectors, which were subsequently used as input in SVM for prediction. The proposed approach was found to perform better than several existing approaches for predicting AMPs, when comparison was made using bench mark dataset.

## Material and Methods

As summarized and demonstrated by a series of recent publications^17^,^18^,^19^,^20^,^21^,^22^, in compliance with Chou’s 5-step rule^23^, to establish a really useful sequence-based statistical predictor for a biological system, the following five guidelines should be followed: (a) construct or select a valid benchmark dataset to train and test the predictor; (b) formulate the biological sequence samples with an effective mathematical expression that can truly reflect their intrinsic correlation with the target to be predicted; (c) introduce or develop a powerful algorithm (or engine) to operate the prediction; (d) properly perform cross-validation tests to objectively evaluate the anticipated accuracy of the predictor; (e) establish a user-friendly web-server for the predictor that is freely accessible to the public. In the following sections, we have described how to deal with these steps one-by-one.

### Dataset

#### Positive

To construct the positive dataset, antibacterial, antiviral and antifungal peptide sequences were collected from publicly available databases (or datasets). Specifically, antibacterial peptides were collected from CAMP, APD3^24^ and AntiBP2; antiviral peptides were collected from CAMP, APD3, LAMP^25^ and AVPpred; antifungal peptides were collected from CAMP, LAMP and APD3. The sequences having non-standard amino acids were then removed followed by removal of redundant sequences, similar to earlier studies^7^,^12^,^26^. Since AMPs are mostly 10–100 amino acids long^1^, sequences having less than 10 amino acids were also excluded from further analysis. A summary of the positive datasets is given in Table 1.

#### Negative

The non-antibacterial and non-antiviral peptides were collected from AntiBP2 and AVPpred respectively. These non-antibacterial and non-antiviral peptides were respectively used as the negative dataset against the antibacterial and antiviral peptides. Further, these non-antibacterial and non-antiviral peptides were considered together as the negative dataset against the antifungal peptides. Similar to the positive dataset, sequences of the negative dataset were also processed. A summary of the negative datasets is also given in Table 1.

### Feature generation

Since the peptide sequences are the strings of amino acids, they need to be mapped onto numeric feature vectors before being used as an input in supervised learning classifiers. In this study, three different categories of features i.e., compositional, PHYC and STRL were considered. In particular, 3 compositional (amino acid composition-AAC, pseudo amino acid composition-PAAC and normalized amino acid composition-NAAC), 3 PHYC (hydrophobicity, net-charge and iso-electric point) and 3 STRL (α-helix propensity, β-sheet propensity and turn propensity) features were considered (Table 2) for prediction of AMPs. The compositional and PHYC features were computed by using the “Peptide” package^27^ of R-software^28^, whereas the STRL features were computed by using the TANGO software^29^ available at http://tango.crg.es/. The TANGO server was first used by Torrent *et al*.^30^ for recognition of AMPs. Furthermore, to know the importance of each feature in predicting the antibacterial, antiviral and antifungal peptides, information gain was computed for all the 66 features [AAC (20) + PAAC (20) + NAAC (20) + PHYC (3) + STRL (3)]. To compute the information gain, the *InfoGainAttributeEval* function available in RWeka^31^ package was used.

### SVM-based prediction

We used SVM for prediction of AMPs because it is a non-parametric (does not make any assumption about the underlying probability distribution of the input dataset) and most widely used supervised learning technique in the field of bioinformatics, attributed to its sound statistical background^32^. The predictive ability of SVM, mainly depends upon the type of kernel function that maps the input data to a high-dimensional feature space, where the observations belong to different classes are linearly separable by a optimal separating hyper plane. In this work, the radial basis function (RBF) was used as kernel, due to its wide and successful application in most of the AMP prediction studies^1^,^9^,^10^,^33^. Further, in RBF kernel, default values of parameters gamma (gamma = 1/number of attributes) and cost (C = 1) were used to train and test the prediction model. The *svm* function available in the *e1071* package^34^ of R-software was used to execute the SVM model. The scaling option was kept as TRUE in *svm* function, while training the model.

### Performance evaluation

We considered different performance metrics *viz.,* sensitivity (*Sn*), specificity (*Sp*), accuracy (*Ac*) and Matthew’s correlation coefficient (MCC) to evaluate the performance of the proposed approach. Since, the conventional formulae of these metrics are not quite intuitive, particularly MCC, Chen *et al*.^35^ derived a new set of equations for the above mentioned metrics based on the Chou’s symbols used in studying protein signal peptide cleavage sites^36^. The new formulae for these metrics are given in equation (1)


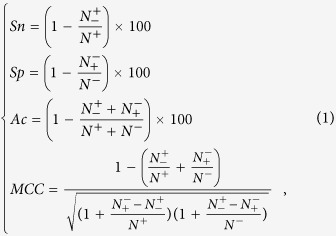


where 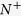
represents the total number of AMPs investigated, 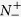
represents the number of AMPs incorrectly predicted as non-AMPs, 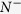
represents the total number of non-AMPs investigated and 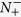
represents the number of non-AMPs incorrectly predicted as AMPs. The formulae given in equation (1) has made the meaning of *Sn, Sp, Ac*, and *MCC* much more intuitive and easier-to-understand, particularly for the meaning of MCC, as concurred by a series of studies published very recently^19^,^20^,^37^,^38^,^39^,^40^,^41^. The above formulae are valid only for the single-label systems, whereas for the multi-label systems, whose emergence has become more frequent in system biology^42^,^43^ and system medicine^22^,^44^,^45^, a different set of metrics is needed as elaborated in Chou^46^.

### Training and validation

In an unbalanced dataset (i.e., the number of AMPs and non-AMPs are not same), machine learning based classifier may produce results biased towards the major class^47^ (having large number of sequences than the other class). Therefore, number of sequences of the major class was kept same as the number of sequences present in the minor class to train the prediction model effectively. Here, sequences of the major class were randomly drawn from the available sequences. Since one random set from major class may not be adequate to judge the generalized predictive ability of the classifier, one thousand random samples (drawn without replacement from major class) were used. Further, in each sample (consists of AMPs and non-AMPs) a 10-fold cross validation^48^ procedure was employed to assess performance of the predictor. Furthermore, to assess the impact of size (number of sequences) of dataset, three datasets with different sample sizes were used (Table 3).

### Comparison with existing methods

Performance of the proposed approach was compared with that of latest AMP prediction tools viz., CAMP^3^, iAMP-2L^7^, EFC-FCBF^12^, EFC + 307-FCBF^12^. The comparison was made by using the Xiao *et al*. benchmark dataset^7^ (http://www.jci-bioinfo.cn/iAMP/data.html). In this dataset, the training set contains 770 antibacterial peptides and 2405 non-AMPs and the test set contains 920 AMPs and 920 non-AMPs. The same datasets have been used by Veltri *et al*.^12^ to evaluate the performance of EFC-FCBF and EFC + 307-FCBF approaches. Further, performances of the methods were compared in terms of area under receiving operating characteristics curve^49^ (AUC-ROC), area under precision-recall curve^50^ (AUC-PR) and MCC. For a binary classifier, recall is same as *Sn* (as defined in equation-1) and precision is defined as 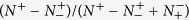.

### Development of prediction server

An online prediction server was also developed using hyper text markup language (HTML) and hypertext preprocessor (PHP), where a developed R-code was executed in the backend upon submission of peptide sequences in the FASTA format. The user can submit single or multiple sequences having only standard amino acid residues. This web server can be used to predict the probabilities with which a candidate peptide sequence can be classified into antiviral, antibacterial and antifungal categories.

## Results

### Performance analysis for predicting the antibacterial peptides

Three different sample sizes (100, 500, 983) were used for prediction of antibacterial peptides. Prediction accuracies for the sample size 983 are given in Table 4, whereas for the sample sizes 100 and 500 accuracies are provided in Supplementary Table S1. It is observed that the prediction accuracies are more precise (low standard error) for the sample size 983 as compared to that of sample sizes 100 and 500. Further, low prediction accuracies are observed with the compositional features alone, whereas 2–6%, ~1%, 2–4% and 4–5% increment in sensitivity, specificity, accuracy and MCC are observed respectively while the compositional, PHYC and STRL features are used together (Table 4 and Supplementary Table S1).

### Performance analysis for predicting the antiviral peptides

For the sample size 738, performance metrics of the proposed approach in predicting the antiviral peptides are given in Table 5, whereas for the sample sizes 100 and 500 accuracies are provided in Supplementary Table S2. It is seen that the prediction models based on the sample size 738 are more stable (low standard error) as compared to those based on sample sizes 100 and 500. Similar to antibacterial peptides, low prediction accuracies are also observed while only compositional features are used, whereas sensitivity, specificity, accuracy and MCC are observed to be increased by 1–3%, 1%, ~1% and 1–3% respectively while all the three features are accounted together (Table 5 and Supplementary Table S2). Besides, it is seen that the accuracies in predicting the antiviral peptides are low as compared to the antibacterial peptides.

### Performance analysis for predicting the antifungal peptides

In case of antifungal peptides, prediction accuracies for the sample size 1383 are given in Table 6 and accuracies for the sample sizes 100 and 500 are provided in Supplementary Table S3. It is observed that the accuracies are more precise for the sample size 1383 as compared that of sample sizes 100 and 500. Similar to antibacterial and antiviral peptides, a decreasing trend in accuracies is observed for all the sample sizes, while PHYC and STRL features are not included in prediction. In particular, sensitivity, specificity, accuracy and MCC are increased by 1–2%, ~1%, ~1% and 1–2% respectively while compositional features are used along with the PHYC and STRL features (Table 6 & and Supplementary Table S3). Furthermore, the accuracies for predicting the antifungal peptides are found higher than that of antiviral peptides and lower than that of antibacterial peptides.

### Feature importance

**Figure 1:**
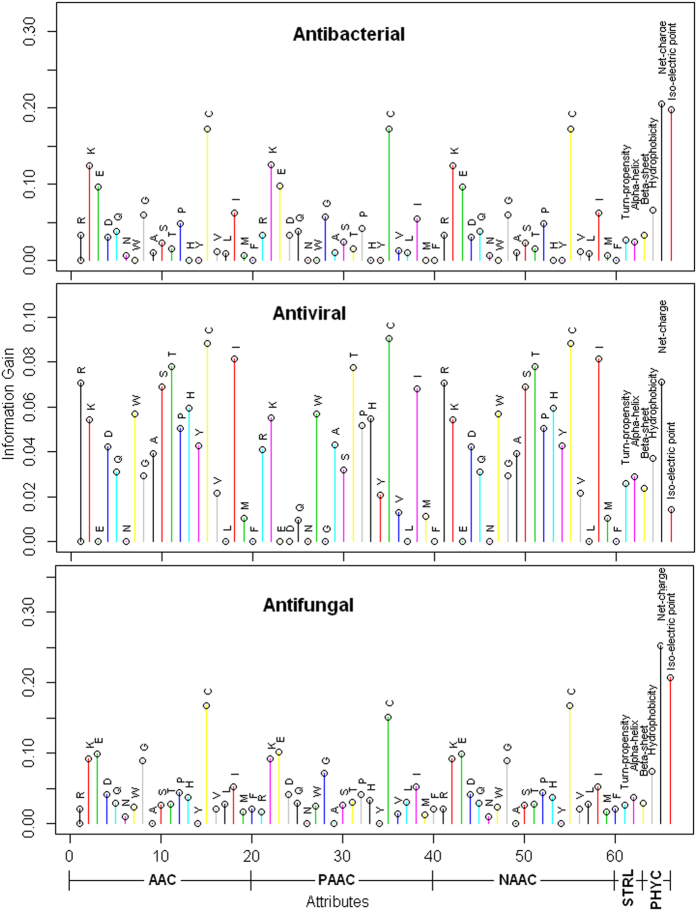
Information gain for all the 66 features [AAC (20) + PAAC (20) + NAAC (20) + PHYC (3) + STRL (3)] in predicting antibacterial, antiviral and antifungal peptides.

Based on top the model (AAC + PAAC + NAAC + STRL + PHYC), information gain for all the features was computed by using the largest sample size and are shown in Fig. 1. From the figure, it can be seen that the values of information gain are almost same for both the AAC and NAAC features. Further, it is observed that the information gain is highest for the feature *net-charge* followed by *iso-electric point*, while predicting the antibacterial and antifungal peptides. On the other hand, highest information gain is observed for the composition of amino acid C, while predicting the antiviral peptides. Furthermore, the STRL features are found less important (low information gain) than that of PHYC features and several compositional features. In particular, values of information gain are seen ≥0.05 for the amino acid compositions K, E. G, P, C and I in case of antibacterial and antifungal peptides, whereas it is ≥0.05 for the amino acid compositions R, K, W, S, T, P, H, C and I in case of antiviral peptides. Besides, values of information gain are observed close to zero for the amino acid compositions {N, W, V, L, M, F, H, Y}, {N, E, L, F} and {A, Y, N} in predicting the antibacterial, antiviral and antifungal peptides respectively. The values of information gain for other amino acids are observed to lie between 0 and 0.05.

### Performance analysis for predicting the AMPs

For prediction of AMPs in general, positive dataset of AMPs was constructed by combining the antibacterial, antiviral and antifungal peptides, whereas negative dataset (non-AMP) was constructed by combining the non-antibacterial and non-antiviral peptides collected from AntiBP2 and AVPpred respectively. Besides, AMPs available in the LAMP were also included in the positive dataset. Finally, a dataset consisting of 5155 AMPs and 1384 non-AMPs was prepared. Similar to antibacterial, antiviral and antifungal, prediction of AMPs was also made with three different sample sizes i.e., 100, 500 and 1383. Moreover, the prediction was made only for the *AAC + PAAC + PHYC + STRL* and *PAAC + NAAC + PHYC + STRL* feature combinations, as little higher accuracies were obtained with these combinations in earlier predictions. The values of different performance metrics (averaged over 10-fold) are given in Table 7. From the table it is seen that the sensitivity, specificity and accuracy are > 90% for all the sample sizes. In addition, the performance of SVM with the above mentioned feature sets were also assessed by using Xiao benchmark training dataset, based on three different sample sizes (100, 500 and 769). The values of different performance metrics (averaged over 10-folds) are given in Table 8. From the table it is observed that the sensitivity, specificity and accuracy are ~94%, whereas for MCC it is ~88%. It is further seen that the prediction accuracies are more precise (low standard error) for the sample size 769.

### Comparative analysis

To further assess the predictive ability as compared to the existing approaches, performance of SVM with *PAAC + NAAC + PHYC + STRL* feature set (we call it *i*AMPpred) was compared with the performances of latest AMP prediction tools, by using Xiao benchmark dataset^7^. The results are given in Table 9. We observed that the accuracies of *i*AMPpred are much higher than that of all the four models of CAMP. In particular, it is observed that the AUC-ROC, AUC-PR and MCC values of *i*AMPpred are ~15%, ~20% and ~30% higher than all the four models of CAMP respectively. Though, *i*AMPpred and iAMP-2L performed at par in terms of MCC, AUC-ROC of *i*AMPpred is observed ~3% higher than that of iAMP-2L. Further, it is seen that the prediction accuracies (AUC-ROC, AUC-PR and MCC) of *i*AMPpred are also higher than that of EFC-FCBF and EFC + 307-FCBF (Table 9).

### Comparison of *i*AMPpred with AntiBP2

**Figure 2:**
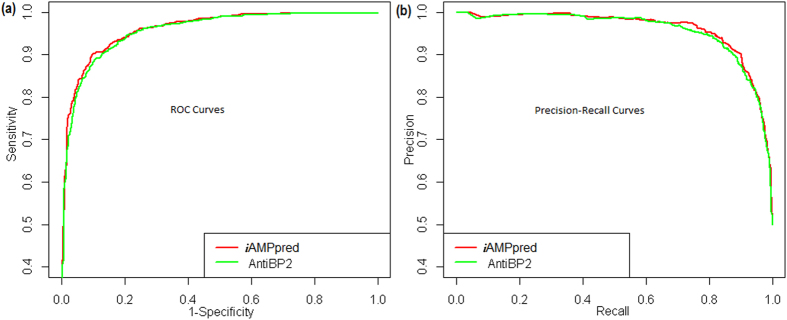
ROC and PR curves of *i*AMPpred and AntiBP2 for the prediction of antibacterial peptides. The performance of *i*AMPpred is found little higher than AntiBP2.

The performance of the *i*AMPpred was also compared with that of AntiBP2 (http://www.imtech.res.in/raghava/antibp2/) by considering the same dataset used in AntiBP2 that contains 999 antibacterial peptides and 999 non-antibacterial peptides. Since 5 sequences in the negative dataset were having non-standard amino acid residues they were excluded from the analysis, and the comparison was made using 999 positive and 994 negative sequences. The ROC and PR curves (averaged over 10-folds) are shown in Fig. 2. We observed that the areas covered under ROC and PR curves for *i*AMPpred are little higher than that of AntiBP2 respectively. This is in accordance with the results presented in Table 4 i.e., the values of performance metrics for PAAC + NAAC + PHYC + STRL feature set (feature set used in *i*AMPpred) are higher than that of AAC feature set (feature set used in AntiBP2).

### Comparison of *i*AMPpred with AVPpred

**Figure 3:**
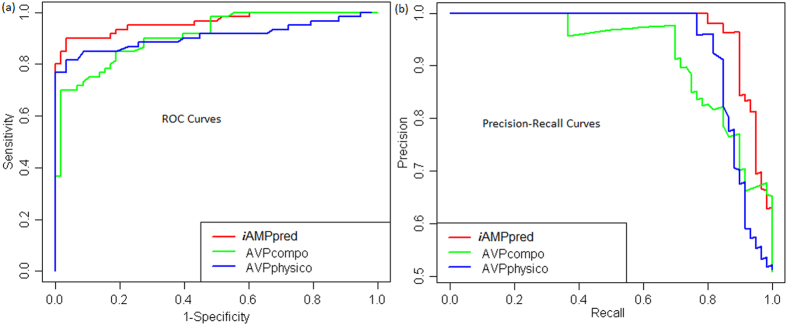
ROC and PR curves of *i*AMPpred and AVPcompo, AVPphysico models of AVPpred for predicting the antiviral peptides. The figure shows that the performance of *i*AMPpred is better than AVPcompo and AVPphysico models of AVPpred.

The performance of *i*AMPpred was further compared with that of AVPpred, by using training [T544(p) + 544(n)] and test [V60(p) + 60(n)] datasets available in AVPpred server (http://crdd.osdd.net/servers/avppred/collection.php?show=dataset). As the accuracies were reported to be higher for AVPcompo and AVPphysico models^10^, they were only considered for comparison. The ROC and PR curves for the test set are shown in Fig. 3. It is observed that the areas covered under both ROC and PR curves for *i*AMPpred are higher than that of both AVPcompo and AVPphysico models. Further, the AVPphysico model performed better than AVPcompo, which is similar to the observation made in Thakur *et al*.^10^.

### Performance analysis of ClassAMP

The performance of ClassAMP, which is meant for predicting the function type of AMPs, was also analyzed by using the Xiao testing dataset. Surprisingly, all the non-AMPs (920) were falsely predicted as AMPs (in any of the three classes) with more than 0.6 probabilities in case of SVM, whereas 915 were falsely predicted as AMPs while RF was used. On the other hand, only 34 and 8 AMPs were falsely predicted as non-AMPs in SVM and RF respectively. This implies that the ClassAMP might be biased towards predicting AMPs. Besides, the accuracies were found higher in *i*AMPpred as compared to that of ClassAMP in predicting the propensity of a peptide sequence as antibacterial, antiviral or antifungal peptides.

### Analysis of organism-specific AMP prediction

Performance of *i*AMPpred was also assessed for prediction of AMPs specific to six different source organisms viz., plants, bacteria, cattle, insects, fishes and amphibians. AMPs for these organisms were collected from APD3 database (1348 AMPs from amphibians, 47 from cattle, 137 from fishes, 341 from insects and 216 from bacteria). The 920 non-AMPs of Xiao testing dataset was considered as the negative dataset against each of the positive datasets. The prediction accuracies in terms of different performance metrics (averaged over 10-fold cross validation) are given in Table 10. Highest accuracy in terms of MCC are observed for amphibians (0.97) followed by cattle (0.94), plants (0.93) and insects (0.92). Interestingly, accuracies for all the organisms are observed >96%, which suggests that the *i*AMPpred is also efficient in predicting the organism-specific AMPs.

### Online prediction server: *i*AMPpred

**Figure 4:**
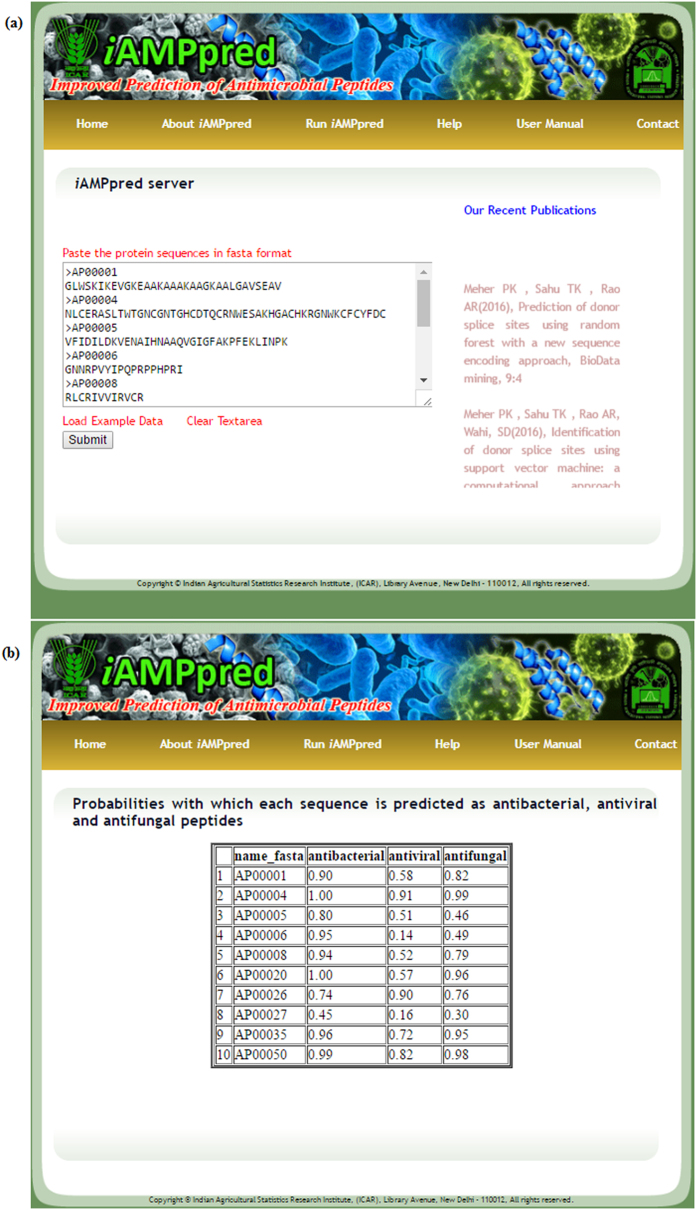
Snapshots of (**a**) server page of *i*AMPpred and (**b**) result page after execution of the program with an example dataset. The results are displayed in a tabular format showing the sequence identifier and the probabilities with which the sequences are predicted as antibacterial, antiviral and antifungal peptides.

An online prediction server “*i*AMPpred” has been developed to predict the propensity of a peptide sequence as antibacterial, antiviral and antifungal peptides. Snapshots of the web pages showing the execution of *i*AMPpred for an example dataset along with the results are shown in Fig. 4. For user guidance with regard to feature generation, prediction method and input-output, links have been provided in the main menu. The sequences with probabilities of being antiviral, antibacterial and antifungal peptides are displayed in the result page. For reproducible research, links to download the trained datasets (http://cabgrid.res.in:8080/amppred/about.html) are also provided. The prediction server is freely accessible at http://cabgrid.res.in:8080/amppred.

## Discussion

AMPs are natural antibiotics gaining attention as an alternative to the chemical antibiotics. Identification and designing of AMPs via wet lab experiments may be resource intensive. Thus, computational identification will supplement to the designing of new antimicrobial agents. This paper presents a SVM-based computational approach that can be used for predicting the effective AMPs with higher accuracy as compared to several existing approaches.

In this investigation, combinations of compositional, PHYC and STRL features were used to map the peptide sequences onto numeric feature vectors that were subsequently used as input in SVM for prediction of AMPs. Though, AAC^9^,^10^ and PAAC^7^,^26^ features have been used in earlier studies, the NAAC feature is used for the first time in our study for AMP prediction. Moreover, α-helix, β-sheet and turn propensity features were also used as they were reported to play an important role in discriminating the AMPs from non-AMPs^30^. Furthermore, Most of the earlier methods were evaluated based on a single dataset of AMPs, collected either from CAMP or APD/APD2 database. On the other hand, the sequences of AMPs used in this study were thought to be more representative as they were collected from several AMP databases. From information gain analysis, *net-charge* was found to be the most important feature followed by *iso-electric point* in predicting the antibacterial and antifungal peptides. On the other hand, the composition of amino acid C was observed to play the most important role in predicting the antiviral peptides. Further, the PHYC features were found to play a more important role than STRL features in predicting the antibacterial, antiviral and antifungal peptides. As far as the compositional features are concerned, amino acids K, P, C and I were found more important as compared to others in predicting the AMPs. On the other hand, the amino acid compositions {N, W, V, L, M, F, H, Y}, {N, E, L, F} and {A, Y, N} were found less important in predicting the antibacterial, antiviral and antifungal peptides respectively.

**Figure 5:**
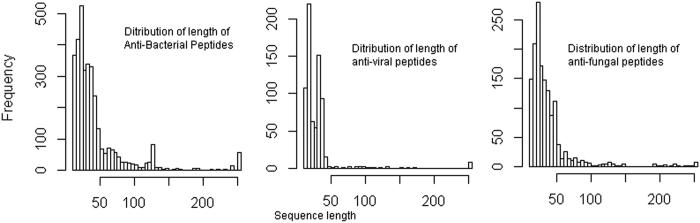
Distribution of length of the sequences in antibacterial, antiviral and antifungal peptides. The antibacterial and antifungal peptides are > 50 amino acids long, whereas most of the antiviral peptides are < 50 amino acids long.

The prediction of antibacterial, antiviral and antifungal peptides was made by using three different sample sizes. Prediction accuracies were found to be more precise for the large sample sizes as compared to that of small sample sizes. Further, accuracies for predicting the antibacterial and antifungal peptides were found higher than that of antiviral peptides. This might be due to the longer sequence length (10–100 amino acids) of antibacterial and antifungal peptides and shorter sequence length (10–50 amino acids) of antiviral peptides (Fig. 5). Besides, PHYC and STRL determinants were found to play a more important role in the prediction of antibacterial peptides as compared to antiviral and antifungal peptides. Since the prediction accuracies (Sn, Sp, ACC) were also found to be higher (>90%) for prediction of AMPs in general (Table 7), the *i*AMPpred is believed to supplement the existing tools for predicting the antibacterial, antiviral and antifungal peptides independently as well as predicting the AMPs in general.

The performance of *i*AMPpred was also compared with that of several state-of-art AMPs prediction methods by using Xiao benchmark dataset. The *i*AMPpred was found to achieve higher accuracies than all the four models of CAMP, which might be due to the use of AAC and PHYC features in CAMP without STRL features. Moreover, the feature extraction in CAMP is based on the reduced alphabet due to which the information might be lost. The features employed in iAMP-2L is the correlated PAAC that constitutes a subset of *i*AMPpred feature set and this could be one of the reasons for the equivalent performance of iAMP-2L with *i*AMPpred. In EFC-FCBF, the evolutionary feature set was constructed and 40 informative features were selected by fast correlation based feature selection (FCBF)^51^ technique, which were then used as input in logistic classifier. The AUC-ROC and AUC-PR of EFC-FCBF were found closer to that of *i*AMPpred, which implies that the evolutionary features are also important in predicting AMPs. The EFC + 307-FCBF is an extension of EFC-FCBF, where 307 more PHYC features were used to train and test the model. Though the accuracy of this model was found at par with the *i*AMPpred, the number of features used in EFC + 307-FCBF (i.e., 347) is much larger than the number of features considered in *i*AMPpred (i.e., 46).

The performance of *i*AMPpred was also compared with the specific tools such as AntiBP2 and AVPpred meant for predicting antibacterial and antiviral peptides respectively. The accuracies of *i*AMPpred was found little higher than that of AntiBP2 but much higher than that of AVPpred. One of the possible reasons for this may be the non-consideration of NAAC, PAAC, STRL features in both AntiBP2 and AVPpred. The accuracy of *i*AMPpred was also found higher as compared to that of ClassAMP with Xiao testing dataset. Besides, *i*AMPpred achieved higher accuracies for organism-specific prediction of AMPs. The developed web server *i*AMPpred is believed to supplement the existing tools/techniques in predicting the AMPs.

## Additional Information

**How to cite this article**: Meher, P. K. *et al*. Predicting antimicrobial peptides with improved accuracy by incorporating the compositional, physico-chemical and structural features into Chou’s general PseAAC. *Sci. Rep.* **7**, 42362; doi: 10.1038/srep42362 (2017).

**Publisher's note:** Springer Nature remains neutral with regard to jurisdictional claims in published maps and institutional affiliations.

## Supplementary Material

Supplementary Tables S1, S2 and S3

## Figures and Tables

**Table 1:** Summary of the positive and negative datasets.

Dataset	Bacterial	Viral	Fungal
Positive	CAMP^3^, APD3^24^, AntiBP2^9^ {3417}	CAMP, APD3, LAMP^25^, AVPpred^10^ {739}	CAMP, LAMP, APD3 {1496}
Negative	AntiBP2 {984}	AVPpred {893}	AntiBP2, AVPpred {1384}

The value inside bracket {} is the number of sequences collected in that category.

**Table 2:** Summary of the feature sets.

Feature category	Features in each category	#Features
Compositional	Amino acid composition (AAC)	20
	Normalized AAC (NAAC)	20
Structural (STRL)	Pseudo AAC (PAAC)	20
	α-helix propensity	1
	β-sheet propensity	1
	Turn propensity	1
Physico-chemical (PHYC)	Iso-electric point	1
	Hydrophobicity	1
	Net-charge	1

**Table 3:** Number of sequences present (sample size) in three different datasets used for prediction of antibacterial, antiviral and antifungal peptides.

Dataset	Bacterial		Viral		Fungal	
	#ABP	#nonABP	#AVP	#nonAVP	#AFP	#nonAFP
1^st^ set	100	100	100	100	100	100
2^nd^ set	500	500	500	500	500	500
3^rd^ set	983	983	738	738	1383	1383

#ABP: Number of antibacterial peptides, #nonABP: Number of non-antibacterial peptides, #AVP: Number of antiviral peptides, #nonAVP: Number of non-antiviral peptides, #AFP: Number of antifungal peptides, #nonAFP: Number of non-antifungal peptides. In all the cases the instances were randomly drawn (without replacement) from the available number of instances present in the respective classes.

**Table 4:** Performance metrics of SVM in predicting antibacterial peptides for the sample size 983.

Features	Performance metrics			
	Sn ± SE	Sp ± SE	Ac ± SE	MCC
AAC + PAAC	91.16 ± 0.71	93.41 ± 0.49	92.29 ± 0.36	0.85 ± 0.007
AAC + NAAC	91.29 ± 0.79	93.44 ± 0.49	92.37 ± 0.45	0.85 ± 0.009
PAAC + NAAC	91.29 ± 0.65	93.37 ± 0.51	92.33 ± 0.37	0.85 ± 0.007
AAC + PAAC + NAAC	91.35 ± 0.69	93.48 ± 0.52	92.41 ± 0.41	0.85 ± 0.008
AAC + PAAC + PHYC + STRL	93.81 ± 0.55	94.96 ± 0.40	94.39 ± 0.35	0.89 ± 0.007
AAC + NAAC + PHYC + STRL	93.87 ± 0.61	94.85 ± 0.39	94.36 ± 0.36	0.89 ± 0.007
PAAC + NAAC + PHYC + STRL	93.86 ± 0.65	94.91 ± 0.38	94.39 ± 0.35	0.89 ± 0.007
AAC + PAAC + NAAC + PHYC + STRL	93.85 ± 0.59	94.98 ± 0.36	94.69 ± 0.38	0.89 ± 0.008

SE: Standard Error.

**Table 5:** Performance metrics of SVM in predicting antiviral peptides for the sample size 738.

Features	Performance metrics			
	Sn ± SE	Sp ± SE	Ac ± SE	MCC
AAC + PAAC	85.60 ± 0.56	90.72 ± 0.61	88.16 ± 0.38	0.76 ± 0.008
AAC + NAAC	85.42 ± 0.58	90.59 ± 0.69	88.00 ± 0.41	0.76 ± 0.008
PAAC + NAAC	85.47 ± 0.61	90.68 ± 0.59	88.08 ± 0.40	0.76 ± 0.008
AAC + PAAC + NAAC	85.49 ± 0.61	90.77 ± 0.62	88.13 ± 0.40	0.76 ± 0.008
AAC + PAAC + PHYC + STRL	88.67 ± 0.56	91.49 ± 0.68	90.08 ± 0.42	0.80 ± 0.008
AAC + NAAC + PHYC + STRL	88.46 ± 0.59	91.57 ± 0.64	90.01 ± 0.39	0.80 ± 0.008
PAAC + NAAC + PHYC + STRL	88.69 ± 0.59	91.49 ± 0.57	90.09 ± 0.34	0.80 ± 0.007
AAC + PAAC + NAAC + PHYC + STRL	88.65 ± 0.65	91.42 ± 0.67	90.08 ± 0.40	0.80 ± 0.008

SE: Standard Error.

**Table 6:** Performance metrics of SVM in predicting antifungal peptides for the sample size 1383.

Features	Performance metrics			
	Sn ± SE	Sp ± SE	Ac ± SE	MCC
AAC + PAAC	90.71 ± 0.29	93.14 ± 0.24	91.93 ± 0.16	0.84 ± 0.003
AAC + NAAC	90.82 ± 0.32	93.22 ± 0.25	92.02 ± 0.19	0.84 ± 0.004
PAAC + NAAC	90.76 ± 0.35	93.16 ± 0.25	91.96 ± 0.23	0.84 ± 0.005
AAC + PAAC + NAAC	90.77 ± 0.32	93.22 ± 0.21	92.00 ± 0.18	0.84 ± 0.004
AAC + PAAC + PHYC + STRL	92.33 ± 0.37	94.36 ± 0.22	93.35 ± 0.22	0.87 ± 0.004
AAC + NAAC + PHYC + STRL	92.32 ± 0.32	94.36 ± 0.23	93.34 ± 0.20	0.87 ± 0.004
PAAC + NAAC + PHYC + STRL	92.25 ± 0.29	94.38 ± 0.25	93.31 ± 0.17	0.87 ± 0.003
AAC + PAAC + NAAC + PHYC + STRL	92.30 ± 0.27	94.41 ± 0.25	93.35 ± 0.18	0.87 ± 0.004

SE: Standard Error.

**Table 7:** Accuracies of the proposed approach for the prediction of antimicrobial peptides.

Feature	Sample size	Performance metrics			
		Sn ± SE	Sp ± SE	Ac ± SE	MCC
AAC + PAAC + PHYC + STRL	100	93.19 ± 2.32	95.13 ± 2.20	94.16 ± 1.56	0.88 ± 0.031
	500	90.50 ± 1.30	93.68 ± 0.91	92.09 ± 0.73	0.84 ± 0.014
	1383	90.60 ± 0.66	92.98 ± 0.44	91.79 ± 0.39	0.84 ± 0.008
PAAC + NAAC + PHYC + STRL	100	92.50 ± 2.62	95.39 ± 2.26	93.95 ± 1.66	0.88 ± 0.033
	500	90.41 ± 1.31	93.77 ± 0.99	92.09 ± 0.75	0.84 ± 0.015
	1383	90.75 ± 0.82	92.94 ± 0.44	91.84 ± 0.40	0.84 ± 0.008

SE: Standard Error.

**Table 8:** Prediction accuracies of the proposed approach in predicting the antimicrobial peptides using Xiao training dataset.

Feature	Sample size	Performance metrics			
		Sn ± SE	Sp ± SE	Ac ± SE	MCC
PAAC + NAAC + PHYC + STRL	100	96.28 ± 1.76	95.58 ± 2.00	95.93 ± 1.30	0.91 ± 0.026
	500	94.46 ± 0.72	93.83 ± 0.91	94.15 ± 0.53	0.88 ± 0.011
	769	94.10 ± 0.61	93.59 ± 0.81	93.84 ± 0.50	0.88 ± 0.010
AAC + NAAC + PHYC + STRL	100	95.88 ± 1.95	95.57 ± 1.97	95.72 ± 1.35	0.91 ± 0.026
	500	94.51 ± 0.81	93.73 ± 0.93	94.12 ± 0.62	0.88 ± 0.012
	769	94.08 ± 0.52	93.63 ± 0.83	93.85 ± 0.49	0.88 ± 0.009

SE: Standard Error.

**Table 9:** Estimates of AUC-ROC, AUC-PR and MCC for different AMP prediction methods based on independent test dataset.

Methods	AUC-ROC (%)	AUC-PR (%)	MCC
CAMP-SVM	64	53	0.43
CAMP-RF	73	76	0.40
CAMP-ANN	80	NA	0.61
CAMP-DA	81	76	0.49
iAMP-2L	95	NA	0.90
EFC-FCBF	96	95	0.73
EFC + 307-FCBF	95	98	0.86
*i*AMPpred	98	99	0.91

Methods which provide continuous prediction values, we reported AUC-PR. Otherwise, “NA” is shown when methods only report a binary (AMP or nonAMP) prediction.

**Table 10:** Performance metrics for *i*AMPpred in predicting organism-specific AMPs.

Source Organism	Sn	Sp	Ac	MCC
Amphibian	98.81	98.26	98.58	0.97
Bacteria	86.19	98.91	96.55	0.88
Plant	93.70	99.02	97.82	0.94
Fish	81.54	99.46	97.24	0.87
Insect	91.69	99.46	96.90	0.92
Cattle	98.33	99.89	98.44	0.94
