# Supplementary material for: Predicting antimicrobial peptides with improved accuracy by incorporating the compositional, physico-chemical and structural features into Chou’s general PseAAC
Source: Sci Rep. 2017 Feb 13;7:42362. doi: 10.1038/srep42362 (PMC5304217; doi:10.1038/srep42362)
Supplement: Supplementary Tables S1, S2 and S3 [file srep42362-s1.pdf]

Predicting antimicrobial peptides with improved accuracy by incorporating the compositional, physico-chemical and structural features into Chou's general PseAAC

Prabina Kumar Meher[1], Tanmaya Kumar Sahu[2], Varsha Saini[3] and A. R. Rao[2,*]

[1]Division of Statistical Genetics, ICAR-Indian Agricultural Statistics Research Institute, New Delhi-110012, India

[2]Centre for Agricultural Bioinformatics, ICAR-Indian Agricultural Statistics Research Institute, New Delhi-110012, India

[3]Department of Bioinformatics, Janta Vedic College, Baraut, Baghpat-250611, Uttar Pradesh, India

Supplementary Table S1. The values of performance metrics of SVM for predicting the antibacterial peptides for sample sizes 100 and 500.

Sample size	Features	Performance metrics			
		Sn±SE	Sp±SE	Ac±SE	MCC
100	AAC+PAAC	93.43±2.50	95.61±1.90	94.52±1.45	0.891±0.029
	AAC+NAAC	93.00±2.64	95.78±1.98	94.39±1.57	0.888±0.031
	PAAC+NAAC	93.30±2.66	95.46±2.08	94.38±1.57	0.888±0.031
	AAC+PAAC+NAAC	93.70±2.11	95.59±2.04	94.65±1.40	0.893±0.028
	AAC+PAAC + PHYC+STRL	97.38±1.48	96.65±1.84	97.02±1.19	0.940±0.024
	AAC+NAAC + PHYC+STRL	97.42±1.49	96.68±1.85	97.05±1.17	0.941±0.023
	PAAC+NAAC + PHYC+STRL	97.68±1.56	96.83±1.68	97.26±1.13	0.945±0.022
	AAC+ PAAC+NAAC + PHYC+STRL	97.69±1.63	96.85±1.68	97.28±1.14	0.946±0.023
500	AAC+PAAC	91.42±1.11	93.95±0.96	92.69±0.72	0.854±0.014
	AAC+NAAC	91.26±1.06	94.21±0.81	92.73±0.62	0.855±0.012
	PAAC+NAAC	91.44±1.15	93.99±1.06	92.72±0.70	0.855±0.014
	AAC+PAAC+NAAC	91.34±1.03	94.10±0.85	92.72±0.58	0.855±0.012
	AAC+PAAC + PHYC+STRL	97.16±0.72	95.38±0.90	96.27±0.61	0.925±0.012
	AAC+NAAC + PHYC+STRL	97.01±0.74	95.22±0.87	96.12±0.49	0.923±0.010
	PAAC+NAAC + PHYC+STRL	97.12±0.64	95.39±0.81	96.26±0.49	0.925±0.010
	AAC+ PAAC+NAAC + PHYC+STRL	97.22±0.72	95.42±0.84	96.31±0.51	0.926±0.010

SE: Standard Error

Supplementary Table S2. The values of performance metrics of SVM for predicting the antiviral peptides for the sample sizes 100 and 500.

Sample size	Features	Performance metrics			
		Sn±SE	Sp±SE	Ac±SE	MCC
100	AAC+PAAC	90.31±2.96	91.12±3.14	90.72±1.87	0.814±0.037
	AAC+NAAC	90.23±3.18	91.03±3.05	90.63±1.88	0.813±0.037
	PAAC+NAAC	90.47±3.14	91.60±2.80	91.04±1.58	0.821±0.032
	AAC+PAAC+NAAC	90.10±3.24	91.35±2.83	90.73±1.63	0.815±0.032
	AAC+PAAC + PHYC+STRL	91.26±3.29	92.18±3.12	91.72±1.84	0.834±0.036
	AAC+NAAC + PHYC+STRL	91.45±3.16	91.91±3.05	91.68±1.79	0.834±0.036
	PAAC+NAAC + PHYC+STRL	91.42±2.93	92.81±2.47	92.12±1.49	0.842±0.030
	AAC+ PAAC+NAAC + PHYC+STRL	91.41±2.76	92.82±2.79	92.12±1.68	0.841±0.033
500	AAC+PAAC	86.66±1.00	90.09±1.11	88.38±0.60	0.768±0.012
	AAC+NAAC	86.68±1.15	90.31±0.96	88.49±0.67	0.770±0.013
	PAAC+NAAC	86.71±1.10	90.40±1.23	88.55±0.77	0.772±0.015
	AAC+PAAC+NAAC	86.49±0.99	90.30±1.02	88.40±0.63	0.768±0.013
	AAC+PAAC + PHYC+STRL	89.27±1.00	90.98±0.94	90.13±0.69	0.803±0.014
	AAC+NAAC + PHYC+STRL	89.23±1.00	90.93±1.05	90.08±0.68	0.802±0.014
	PAAC+NAAC + PHYC+STRL	89.42±1.12	91.10±1.19	90.26±0.64	0.805±0.013
	AAC+ PAAC+NAAC + PHYC+STRL	89.46±0.98	91.08±1.13	90.26±0.69	0.806±0.014

SE: Standard Error

Supplementary Table S3. The values of performance metrics of SVM for predicting the antifungal peptides for the sample sizes 100 and 500.

Sample size	Features	Performance metrics			
		Sn±SE	Sp±SE	Ac±SE	MCC
100	AAC+PAAC	93.09±2.38	95.44±2.31	94.27±1.64	0.886±0.033
	AAC+NAAC	93.58±2.33	95.28±1.91	94.43±1.47	0.889±0.029
	PAAC+NAAC	93.24±2.49	95.44±1.90	94.34±1.50	0.887±0.030
	AAC+PAAC+NAAC	93.41±2.28	95.35±1.85	94.38±1.37	0.888±0.027
	AAC+PAAC + PHYC+STRL	94.78±2.02	95.80±1.98	95.29±1.41	0.906±0.028
	AAC+NAAC + PHYC+STRL	94.97±2.31	95.64±1.91	95.31±1.40	0.906±0.028
	PAAC+NAAC + PHYC+STRL	95.19±2.14	95.99±1.87	95.59±1.25	0.912±0.025
	AAC+ PAAC+NAAC + PHYC+STRL	95.20±1.97	96.00±1.96	95.60±1.34	0.915±0.027
500	AAC+PAAC	91.50±1.09	93.60±0.94	92.55±0.68	0.851±0.014
	AAC+NAAC	91.28±1.07	93.51±0.88	92.39±0.61	0.848±0.012
	PAAC+NAAC	91.53±1.00	93.65±1.05	92.59±0.73	0.852±0.015
	AAC+PAAC+NAAC	91.59±0.86	93.61±0.95	92.60±0.61	0.852±0.012
	AAC+PAAC + PHYC+STRL	93.08±0.86	94.47±0.78	93.78±0.53	0.876±0.011
	AAC+NAAC + PHYC+STRL	92.93±0.93	94.48±0.83	93.71±0.62	0.874±0.012
	PAAC+NAAC + PHYC+STRL	92.90±0.78	94.50±0.81	93.70±0.54	0.874±0.011
	AAC+ PAAC+NAAC + PHYC+STRL	92.93±1.06	94.57±0.78	93.75±0.64	0.875±0.013

SE: Standard Error
